# Evolution of Select Epidemiological Modeling and the Rise of Population Sentiment Analysis: A Literature Review and COVID-19 Sentiment Illustration

**DOI:** 10.3390/ijerph19063230

**Published:** 2022-03-09

**Authors:** Talal Daghriri, Michael Proctor, Sarah Matthews

**Affiliations:** 1 Department of Industrial Engineering, Jazan University, Jazan 45142, Saudi Arabia; 2 Department of Industrial Engineering & Management Systems, University of Central Florida, Orlando, FL 32816, USA; michael.proctor@ucf.edu; 3 Interdisciplinary Modeling and Simulation Program, University of Central Florida, Orlando, FL 32816, USA; sarah.matthews@Knights.ucf.edu

**Keywords:** pandemics, vaccine hesitancy, social media, social networks, literature review

## Abstract

With social networking enabling the expressions of billions of people to be posted online, sentiment analysis and massive computational power enables systematic mining of information about populations including their affective states with respect to epidemiological concerns during a pandemic. Gleaning rationale for behavioral choices, such as vaccine hesitancy, from public commentary expressed through social media channels may provide quantifiable and articulated sources of feedback that are useful for rapidly modifying or refining pandemic spread predictions, health protocols, vaccination offerings, and policy approaches. Additional potential gains of sentiment analysis may include lessening of vaccine hesitancy, reduction in civil disobedience, and most importantly, better healthcare outcomes for individuals and their communities. In this article, we highlight the evolution of select epidemiological models; conduct a critical review of models in terms of the level and depth of modeling of social media, social network factors, and sentiment analysis; and finally, partially illustrate sentiment analysis using COVID-19 Twitter data.

## 1. Introduction

### Brief Background

Pandemics negatively impact the social behavior of individuals, national economies, and the vitality of the global economy [1]. Pandemic spread predictions, public health protocols, and vaccine adoption models are critical for advising governments, non-government organizations, heads of households, and individuals on behaviors and policies that may reduce their risk and slow the spread of the disease [2]. At the 2020 onset of COVID-19 in the United States of America, model guidance for “fifteen days to slow the spread” locked the nation down [3]. Health protocols such as social distancing, mask wearing, and hand sanitizing led to an initial drop in cases [4,5]. By June 2021, with as many as one in five Americans expressing vaccine hesitancy, vaccine adoption rates slowed at around 50% of the population fully vaccinated [6,7]. With the onset of the Delta variant and corresponding rise in infections, hospitalizations, and deaths, a new surge in vaccinations occurred, though far less than hoped [8]. As a result of unfilled expectation, vaccine mandates were issued which have been met with widespread resistance as to their scope and appropriateness [9,10,11]. While vaccine hesitancy is not a new phenomenon, vaccine hesitancy has the potential of prolonging the longevity of a pandemic and, depending on whether or not the virus mutates into an endemic state, may even enable re-emergence [12].

Clearly government-imposed protocols and policies, no matter how well intended, degrade over time due to mounting social pressures and inevitable civil disobedience [13]. Given significant errors in original model predictions and policies [14], the question arises: what is the state of incorporation of sentiment analysis in epidemiological modeling, and what are its advantages and limitations?

We lay a foundation for discussion of these questions with a literature review of 34 selected models listed and characterized in Appendix A-Model Summary while providing a critical review of each model in terms of the level and depth of modeling of social media factors and sentiment analysis.

## 2. Materials and Methods

Our review used a methodological approach in which 34 selected distinct models typed in the following categories: Epidemiological State Model Foundations, Epidemiological Statistical Forecast Models, Theoretical Interventions Models and Approaches, Agent-based Models and Multiagent Systems Models, and Artificial Intelligence and Hybrid Models. More recent models supplement traditional approaches with information from social media (SM) or social networks (SN). Social media and social networks are varied based on the methodology of the calibration with models, where some models trace GPS records or calls through social networks techniques in order to study public mobility behavior effects during pandemics, while other models are able to analyze population sentiments through their tweets, posts, or short message service (SMS). Sentiment analysis can be performed for the social media platforms that provide opinions and comments, such as Twitter and Facebook. We discuss 34 classifications of models with descriptions of their advantages and limitations as well as whether the sentiment analysis and machine learning approach were able to capture the real opinions of the population about the COVID-19 vaccines. We conducted the sentiment analysis for Twitter users in the USA to describe their attitude and opinions about COVID-19 vaccination during the Delta variant surge.

### 2.1. Epidemiological State Model Foundations

**Figure 1:**
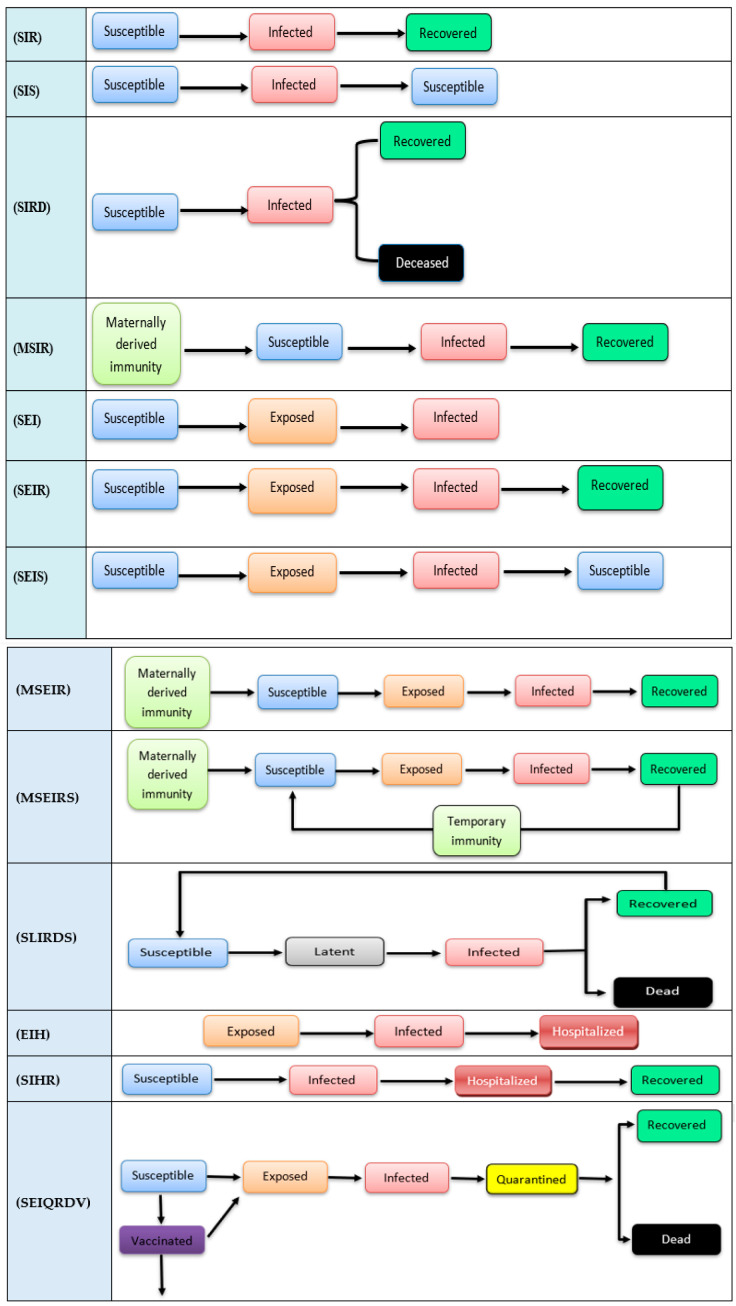
SIR and twelve of its descendant models.

Epidemiological state data observed over time may be used to generate mathematical relationships for estimating state-to-state transitions within the population using assumptions about the disease process, social mixing, public health policies and other inputs [15]. Developed in the 1920s, Kermack and McKendrick’s (1991) Susceptible, Infected, Recovered (SIR) model [16] may be considered the grandfather of epidemiological state modeling (Figure 1). SIR represents population states of susceptible (S), infected (I), or recovered (R) as the disease progresses through the population (Cooper, Mondal and Antonopoulos, 2020). SIR models for person-to-person transmitted diseases confer immunity after infection in contrast SIS (susceptible, infected, susceptible) does not confer immunity [17]. A total of 12 of our 34 models are descendants of the SIR model and are illustrated in Figure 1 below. These include: Susceptible, Infected, Recovered (SIR); Susceptible, Infected, Susceptible (SIS); Susceptible, Infected, Recovered, Deceased (SIRD); Maternally derived immunity, Susceptible, Infected, Recovered (MSIR); Susceptible, Exposed, Infected (SEI); Susceptible, Exposed, Infected, Recovered (SEIR); Susceptible, Exposed, Infected, Susceptible (SEIS); Maternally derived immunity, Susceptible, Exposed, Infected, Recovered (MSEIR); Maternally derived immunity, Susceptible, Exposed, Infected, Recovered, Susceptible (MSEIRS); Susceptible-Latent-Infected-Recovered-Dead-Susceptible (SLIRDS); Exposed, Infected, Hospitalized (EIH); Susceptible, Infected, Hospitalized, Recovered (SIHR); and Susceptible, Exposed, Infected, Quarantined, Recovered, Dead, Vaccinated (SEIQRDV). Each box represents a state of disease which is represented by a set of mathematical rules as people move from state to state. Epidemiological state models have the advantage of flexibility when being built, provide the possible transitions states for the proposed study, and provide a clear exploration and sequence for population states during pandemics. On the other hand, one limitation is that it does not include the public behavior or the human factors that play a significant role in the disease spreading rate and following the health interventions protocols, which affect the models’ prediction accuracy.

In our review, the SEI, EIH, and SIHR models were calibrated with social media for media awareness programs to explore the media effects on public behavior during pandemics which impact the disease spread rates.

### 2.2. Epidemiological Statistical Forecast Models

In our review, 5 models fell within the Epidemiology Statistical Forecast Models category, where statistical forecasting models use the currently recorded data of infected, deaths, recovered cases, and so on to forecast the future rates of cases. They include the Differential Equations Leads to Predictions of Hospitalizations and Infections (DELPHI); Auto-regressive integrated moving average (ARIMA); Los Alamos National Laboratory COVID-19 forecasting using Fast Evaluation and Estimation (LANL COFFEF): John Hopkins model COVID-19 prediction (JHU COVID-19 prediction); Susceptible, Exposed, Infected, Quarantined, Recovered, Dead, Vaccinated forecasting (SEIQRDV.F). Epidemiological Statistical Forecast models build on the aforementioned state models project trends into the future but typically do so based solely on extending statistical trends discovered in the data [18]. In the current COVID-19 pandemic, the Johns Hopkins Coronavirus Resource Center (https://coronavirus.jhu.edu/data, accessed on 9 December 2021) [19] is well known for tracking epidemiological state data and reporting various statistical trends of interest to decision makers.

**Figure 2:**
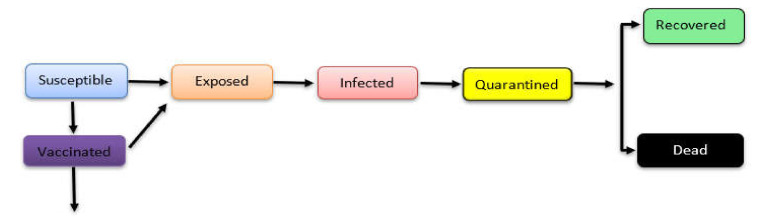
SEIGRDV.F State Model Base (adapted from [20]).

Of the Statistical Forecast models listed, the SEIQRDV.F model (Figure 2) includes a vaccination state [20], where the model studies and analyzes the impact of vaccination step on the pandemic spread trends among the population. Additionally, the SEIQRDV.F model includes additional rate parameters that help predict the effects of vaccines on the disease spread. However, in our review, SEIQRDV.F was not calibrated with SN or SM, but we included it due to the added vaccination feature in the model. Only the ARIMA model within this category was calibrated with SM using Google and Twitter as their data source. The Epidemiological Statistical Forecast Models can provide more accurate predictions compared to the state models as they involve the demographical and/or geographical date. Thus, they can be useful to provide prediction for the results at any place or location. However, these models cannot generate individuals’ behavior effects on the pandemic trends because they do not involve the differences among people, such as reasons behind performing or ignoring the action, and the individual opinions.

### 2.3. Theoretical Intervention Approaches and Models

Statistical forecast models track and extend data rates but do not necessarily explain radical changes or differences in rates such as the sudden increases and fluctuations in infections between communities or nations. Theoretical intervention approaches attempt to project disease outcomes beyond simple statistical trend extrapolation by modeling the underlying dynamics of disease transmission, population behavior and vulnerability, and preventive interventions [21]. Modeling underlying dynamics of pandemics is not new [22]. System dynamics models are applied across a broad spectrum of applications including pandemics, sexual behavior, smoking, exercising, and the use of seat belts [23]. A fundamental objective of applying systems dynamics for pandemic application is to model, simulate, and quantify interventions and outcomes with the hope of limiting disease spread and impacts [24,25]. During the COVID-19 pandemic, objectives included identifying behavioral intervention measures to “slow the spread” [3] and gain time to develop vaccines to counter the disease.

At least three theoretical intervention models that attempt to capture the dynamics of human behavior in light of life-threating events have been put forth: the Health Belief Model (HBM), [26], the Theory of Planned Behavior (TPB) [27], and the Protection Motivation Theory (PMT) [28]. These approaches may be applied to state models or integrated together for deeper work, such as integrating TPB theory and PMT theory to gain insights into both behaviors and intentions of individuals [29].

**Figure 3:**
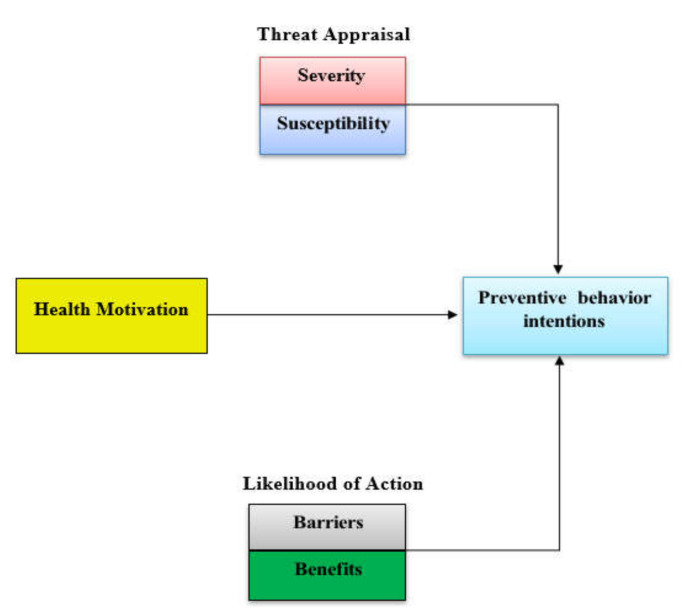
HBM model (Figure adapted from Lipman & Burt (2017)) (adapted from [35]).

Still in use today, the grandfather of theoretical intervention models is the Health Belief Model (HBM) (Figure 3) developed in the 1950s by health, medical, and social scientists in the United States of America [26]. HBM attempts to explain the human behavioral response to outbreaks and associated health protocols. Influences on human behavior included demographics and psychological variables acting through perceived susceptibility to the health threat, perceived severity of the health threat, health motivation, perceived benefits to taking the prescribed action, and perceived barriers to taking actions. For the COVID-19 pandemic, an example of a perceived barrier to health protocols is the tendency for individuals to stay indoors, where viruses may linger, during cold weather [30]. In Figure 3, one can see the rise in infections in the United States of America as the cold weather season began in November 2020. Another major limitation of HBM is its failure to consider conflicting benefits. An excellent example of a conflicting benefit is the desire to congregate with family and friends during traditional holidays and experiencing the love and friendship of those dear to one another. That benefit conflicts with the benefit of the COVID-19 social-distancing and mask-wearing health protocols. As many will choose the former benefit and risk violating the COVID-19 health protocol and foregoing its benefit, the natural result is a spike in virus infections subsequent to the holidays. Spikes can be seen in Figure 3 in the United States of America and in India subsequent to their traditional holidays [31,32].

The Theory of Planned Behavior (TPB), in its simplest form, involves individuals evaluating a proposed behavior. If the individuals feel that their actions are important to others and wish to do so, then they are more likely to carry out the proposed behavior. A high association of behaviors and subjective norms with behavioral intent and actual behavior has been verified in several studies, such as COVID-19 pandemic studies [33,34]. Limitations of the model include the need to design data collection methods suited to individuals and relate those actions to impacts of the disease.

PMT draws on factors similar to HBM including perceived severity of a threatening event, perceived probability of the occurrence or vulnerability, efficacy of the recommended preventive behavior, and perceived self-efficacy [28]. Moreover, these factors may be grouped more generally in terms of appraisal and the coping appraisal. Threat appraisal involves the perceived vulnerability, severity, and level of fear arousal, while the coping appraisal involves response efficacy, self-efficacy, and perceived response-cost [36,37]. Complicating interventions such as controlling weight to prevent heart disease or stroke is the possibility that the intervention may have a side effect that causes another problem [29]. Furthermore, interventions may involve a secondary intervention such as using a medicine to reduce the risk of heart attack [38].

Moreover, the theoretical intervention approaches and models explore the causes and effects of public behaviors and intentions on the pandemic spread, as they explain and study the factors that motivate people to follow the health interventions, or the barriers that push them to not follow the protocols. So, they generate a clear description for the relationships between people and interventions, and these relationships are affected by the personal perceptions.

In our review, only the HBM used SM for calibration on the Facebook platform as a data source.

### 2.4. Agent-Based and Multiagent Systems Modeling

Agent-based and multiagent systems attempt to model individuals (i.e., agents) and influencers of individuals in a simulated community.

Going beyond latent factors, agent-based and multiagent systems share basic artificial intelligence techniques by modeling “person(s), firm(s), machine(s), or software” that generate the actions or interactions [39].

Potentially within the real world using the hybrid techniques discussed below [40], “Agent-based modeling (ABM) explore(s) how the interactions of heterogeneous individuals impact on the wider behavior of social/spatial systems” [41] with “autonomous and pro-active actors, such as human-centered systems” [42]. Modeling micro-interactions and emergent macroscale behavior as autonomous entities enables each agent to assesses its situation and makes decisions based on a set of rules [43] suited to itself [40]. In pandemic-spread studies, an ABM approach “can capture the dynamics of disease spread combined with the heterogeneous mixing and social networks of agents” [44]. By characterizing the disease transmission rates, agents, and their environment, it is possible to generate real scenarios for the pandemics trends [45]. Thus, this kind of model can take the individual differences among population and explore how these differences affect the opinion and final decision regarding following the healthy interventions and talking a vaccine. To elaborate, HBM explores the potential cause-and-effect relationships between the population and the health interventions, benefits and barriers to apply these interventions, while agent-based models explore how the individual differences among agents make them incline to the benefits side or to the barriers side to follow the interventions. The agents can make a decision about following the healthy action but can also ignore it or delay it.

Multiagent systems are notionally more appealing for pandemic applications then a basic ABM approach [46] by addressing some ABM limitations [47,48,49]. Advantages include the recognition and implementation of levels of social or organizational control over entity self-regulation or self-determination, representation of availability and access to channels of communications, and the nature, content and level of trust of information disseminated on those channels [46]. For pandemic spread studies, multiagent systems may model communications, cooperation, collaboration or competitiveness interactions between heterogeneous agents to achieve a decision or goal [42,50].

In our review, there are 7 agent-based and 1 multiagent model pandemic applications discussed in the literature. The Coupled Contagion Dynamics of Fear and Disease (CCDFD) model [51] contains seven states derived from the SIR model but models disease dynamics and fear as two interacting contagion processes. The Social Distancing (SD) model tests effects of different levels of social distancing policies on the disease spread [52]. The COVID-19 Agent-based Simulator (COVASIM) model [53] considers the effects on COVID-19 epidemiology of multiple non-pharmaceutical interventions using agents with “country-specific demographic information on age structure and population size” that interact within “realistic transmission networks in different social layers, including households, schools, workplaces, long-term care facilities, and communities”. Additionally, “age-specific disease outcomes, and interhost viral dynamics, including viral-load-based transmissibility” lend greater fidelity to disease dynamics. Silva et al., 2020 developed the COVID-19 agent-based simulation (COVID-ABS) model from SEIR model using agents organized into persons, houses, business, government, and health systems that communicate and interact with each other. The model emphasized social distancing policies on the COVID-19 pandemic, including: “(1) do nothing, (2) lockdown, (3) conditional lockdown, (4) vertical isolation, (5) partial isolation, (6) use of face masks, and (7) use of face masks together with 50% of adhesion to social isolation” [54]. Li and Giabbanelli’s 2021 COVID-19 Agent-based Simulator (COVASIM) and Vaccination model includes vaccination as an additional intervention. The model focused on virus growth rates for different vaccination scenarios, different nonpharmaceutical interventions, and different vaccine efficacy and compliance levels [55]. Vyklyuk et al., 2021 developed Multi-agent Susceptible, Infected, Recovered DMAS-SIR models to assess how COVID-19 spread through public transportation, supermarkets, colleges, universities, gyms, churches, and parks. Factors include “incubation period, people’s keeping a safe distance when moving, simulated quarantine, isolation.” [50].

FM and UT COVID-19 SD were the two models which included social network in their calibration, using mobile phone calls and GPS traces respectively as their data sources [56,57].

### 2.5. Artificial Intelligence and Hybrid Models

Artificial Intelligence and hybrid models include advanced artificial intelligence or a combination of real-world, real-time data-collection techniques to fit specific applications. There are 6 identified approaches we reviewed. First, the Youyang Gu COVID-19 (YYG) model [58,59,60] is based on the SEIR state model to simulate the COVID-19 epidemic in a given area of concern. Machine learning training methods aim to minimize the difference between the predicted and actual outputs such as estimates of pandemic deaths. Secondly, the deep transfer learning (DTL) model [61] focuses on real-time remote surveillance and monitoring of mask-wearing protocols at points of interest. Thirdly, the University of Virginia Biocomplexity Center PatchSim COVID-19 [62] hybrid model is an extension for the SEIR model [63] that includes mobility tracing [64]. Fourth, the Institute for Health Metrics and Evaluation COVID-19 (IHME COVID-19) model also builds on the SEIR model to assess whether COVID-19 case rates would exceed the capacity of healthcare facilities [65]. The hybrid modeling approach integrates demographic forecasts, agents, and a social distancing policy based on individuals’ phone mobility traces. Forecasts include demand for hospital care, daily and cumulative cases and deaths due to COVID-19 by location [66]. Fifth, the Massachusetts Institute of Technology COVID-19 (MIT University COVID-19) model is also based on the SEIR model and uses a neural network (“Model quantifies the impact of quarantine measures on COVID-19′s spread”, 2020) to assess the effectiveness of quarantine steps and to help forecast the spread of the virus [67]. Dandekar and Barbastathis, 2020 improved the SEIR model by training a neural network to detect the number of infectious individuals that are under quarantine and therefore no longer transmit the infection to others [68]. Sixth, we included a Twitter vaccination analysis model, which used Twitter as a data source, where users’ tweets are collected and analyzed to under-stand public opinions regarding vaccination, where the model quantifies the COVID-19 vaccine hesitancy among nations. Artificial intelligence and hybrid models have the advantage of involving more than one model, which reduces the limitations of each model and increases the efficiency of the analysis and its predictions. Additionally, it may include a technological tool that captures additional features, such as the DTL model.

## 3. Social Media and Epidemiological Modeling

The numerous models clearly demonstrate that the transmission of a disease is influenced directly by people’s behaviors and social interactions [69], where the lockdown, health practice, and social distancing interventions show significant results in flattening the disease spread curve such as lockdown and a testing model [70] and SD model [55]. Historically, governments used conventional broadcast media and various forms of social media to promote healthy behavior and communicate infection diseases outbreak alerts, public health protocols, vaccination programs, etc. [71,72]. Social media is considered an influential factor on individual behavior and provides widespread dissemination of information to citizens in a timely manner [73]. Thus, modeling social networks attempts to incorporate the influence of the these social factors in modeling predictions [74]. Additionally, researchers may find social media datasets useful for analysis purposes. Given the natural language nature of social media in all its forms, data-mining questions include: how are various epidemiological issues represented and conveyed in social media? Once translated from various social media vernaculars, how does information impact decision-making and risk management behavior [75]? A major caveat to social media is that user-generated knowledge about infectious diseases is not always exact or useful and may involve rumors, misinformation, and theories on conspiracy [76]. Thus, it is important to thoroughly study the use of social media and its influence on the level of understanding and affective reactions of the public to epidemiological concerns [77]. For example, EIH models can study the effect of an awareness program on public opinions regarding interventions. Other models use Twitter users’ tweets and Facebook posts to analyze users’ opinions about a specific topic. Social networks were used in other studies to show how phone calls or GPS records are used to trace human mobility and understand behavior regarding the interventions and quantify how that affects disease spread. Regarding fake Twitter and Facebook accounts, they do not represent any sufficient effects on the results, where they are too low compared to the real users’ accounts. To confirm, most of the previous studies that analyzed Twitter population opinions proved that fake accounts have no significant effects on the analysis results.

### 3.1. Epidemiology State Models and Social Media

Tchuenche and Bauch, 2012; Cui et al., 2008; Sooknanan and Comissiong, 2020; and Liu et al., 2007 integrated social media with Exposed, Infected, Hospitalized (EIH) state models [78,79,80,81]. Only when infected case numbers are seen as significant does the public behavior change to protect themselves [82]. Thus, the social media impact was added as a mathematical function to the model to show how social media can utilize fear points as a motivation to push people to follow the interventions through releasing the increase in the number of infected cases and the high risk of being infected. So, their findings indicate that incorporating social media significantly impacts public behavior to reduce the rates of suspectable and infected cases.

### 3.2. Statistical Prediction Models and Social Media

Samaras, García-Barriocanal, and Sicilia, 2020 used Twitter and Google data with the ARIMA model to forecast infections [83]. The aim of their study was to collect evidence on which data source, Twitter or Google, leads to better results. Weekly influenza data over a 23 week period was obtained from Google and Twitter for Greece and placed in the ARIMA model which forecasted future weekly outcomes. These outcomes were compared with influenza data from the European Center for Disease Prevention and Control for validation purposes. The results of this study indicated that Twitter data were significantly better than Google data.

### 3.3. Theoretical Interventions Models and Social Media 

Raamkumar, Tan, and Wee, 2020 integrated social media into a Health Beliefs Model [84]. Facebook users posted their comments on COVID-19 posts from three agencies: the Singapore Health Ministry, the Public Health in England (PHE), and Centers for the Prevention of Diseases. Comments made on social distancing were labeled manually by a yes/no flag in all four HBM constructs. A neural network trained and validated text classification using 16,752 responses. The classification model was able to predict beliefs, trends, and behaviors toward following healthy interventions.

### 3.4. Agent-Based and Social Media and Social Networks

#### 3.4.1. Frias-Martinez (FM) Model

Frias-Martinez et al., 2011 [57] discuss techniques framed by a previous SIR model study by Cruz-Pachecon et al., 2009 [85] that integrates agent-based models with social networks and captures aspects of human mobility from call records. The method was used to study the 2009 H1N1 epidemic in Mexico, to evaluate the effect of government interventions on virus spread, and to predict people’s intentions to move. The model not only represented the mobility behavior and social patterns of a community but also how these patterns shifted over time. These improvements are important because they offer a novel approach to ABM simulations based on real actions, which focuses on the agents’ mobility and social networks.

#### 3.4.2. University of Texas at Austin’s (UT COVID-19-Social Distancing) Model

The UT COVID-19 SD University of Texas at Austin model predicts COVID-19 case rates based on the SEIR model [56]. UT COVID-19 SD quantifies the effects of social distancing intervention on disease spread through mobility traces focusing on the first wave of COVID-19 deaths in the United States of America [56]. UT COVID-19 SD incorporated analyzed obtained data from mobile-phone GPS traces, similar to Frias-Martinez et al., 2011, by inferring locations of mobile phones using SafeGraph. According to Woody et al., 2020 [56], “This data source quantifies two main types of distancing behavior: (1) changes in visitation patterns to public places like restaurants, bars, schools, parks, pharmacies, grocery stores, etc.; and (2) time spent at home versus at work” [86].

### 3.5. Hybrid Models and Social Media

#### Twitter and Vaccination Prediction Model

In recent years, social media platforms became a significant resource of data and analysis. Content within tweets provide data to predict public behavior, beliefs, or opinions regarding specific events, personalities, or subjects. In terms of epidemiology, Sattar and Arifuzzaman, 2021 [87] used Twitter and machine-learning algorithms to capture and identify public sentiment toward vaccines based on around 1.2 million tweets collected across five weeks of April–May 2021. Twitter data were then used to project that around 62.44% and 48% of the US population will have at least one dose of vaccine and be fully vaccinated, respectively, by the end of July 2021. As of July 31 2021, the actual one-dose vaccination rate was 57.% (164.45 million) and 49.53% were fully vaccinated (190.98 million) [88]. Therefore, this hybrid model involves an agent-based modeling approach and machine-learning approach, where Twitter users (agents) posted their opinions about vaccination, and a machine-learning approach was used to extract public opinions and classify them. Thus, the model can generate valuable results in a short time for the selected location and time.

## 4. Illustration of Twitter Sentiment Data

**Figure 4:**
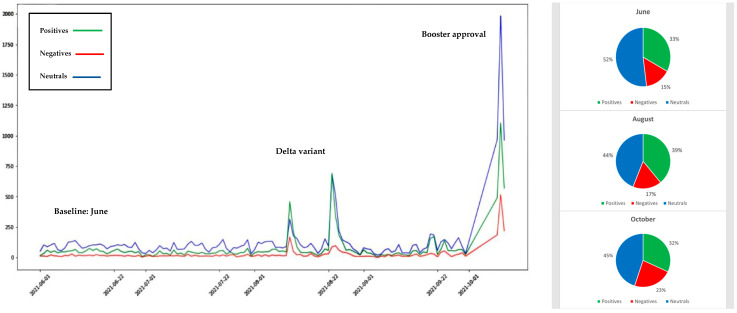
Sentiment analysis results of tweets related to Pfizer vaccine.

**Figure 5:**
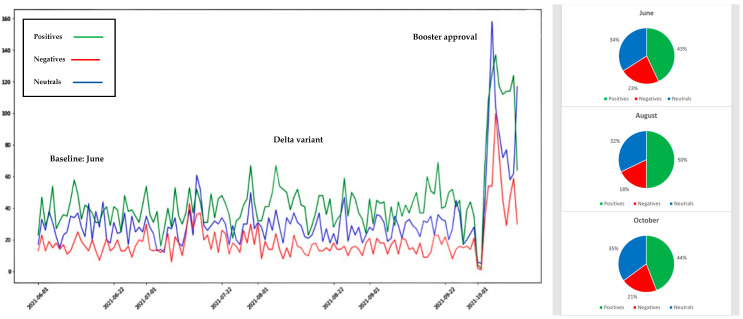
Sentiment analysis results of tweets related to Johnson and Johnson vaccine.

**Figure 6:**
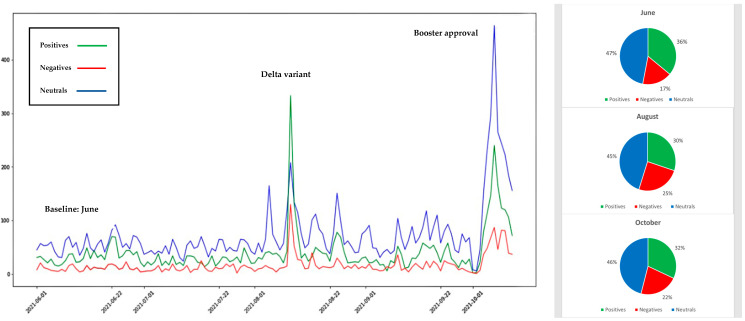
Sentiment analysis results of tweets related to Moderna vaccine.

To illustrate the Sattar and Arifuzzaman, 2021 approach, machine learning and sentiment analysis approaches were used to quickly and effectively measure vaccine hesitancy in the United States of America for the period June 2021 to October 2021. During this period, Twitter chatter reflected user changing sentiment toward vaccines in light of the Delta variant spread and booster shot and child vaccination approval. We extracted and analyzed Twitter sentiment datasets totaling 59,534 tweets for the three vaccines used in the USA (Pfizer, Moderna, and Johnson and Johnson). The results shown in Figure 4, Figure 5 and Figure 6 reflect the change in public sentiment from the June baseline to the August Delta variant peak, and booster shot and child vaccination approval issued in October 2021. Overall, Pfizer had more tweets than J&J and Moderna. From the June baseline to the August Delta variant peak, vaccine positivity increased. CDC data reflected that positivity with an increased vaccination rate. On the other hand, from August to October, Pfizer vaccine positivity dropped and negativity increased. Overall, Pfizer vaccine negativity increased from 15% of the June baseline of the population to 23% in October. Moderna had the second largest population of tweets. Interestingly, the positivity of tweets on Moderna increased and negativity decreased from August to October, though the final negativity toward Moderna at 22% was similar to Pfizer’s 23% negativity. Though dealing with the smallest population of tweets, from August to October, Johnson and Johnson vaccine positivity dropped and negativity increased, though negativity decreased from the June baseline. The October negativity of 21% was also similar to Moderna’s 22% and Pfizer’s 23%. One may consider vaccines a technology. In that light, the 21–23% negativity rate exceeds the 16% laggard technology adoption rate identified by Rogers (1995) [89] and highlights the fear aspect endemic to vaccine hesitancy that is not present in technologies considered by Rogers.

## 5. Discussion and Limitations

In order to focus on COVID-19 vaccination, we extended Sattar and Arifuzzaman’s model, which used Twitter to extract public sentiments about taking the vaccine. Our study is inclusive of data during the Delta variant wave, whereas previous studies did not include this timeframe. We utilized Twitter sentiment analysis to explain the public intentions and opinions toward vaccination during the Delta variant surge. In addition, we compared the sentiment analysis results to CDC datasets which show significant correlations. This model involves agent-based modeling, social media, and a machine-learning approach. It shows how Twitter outputs were useful to provide efficient insights into vaccination prediction studies and how the Delta variant was a strong motivation for increasing vaccination rates in the USA. Comparing to the HBM model, the extracted sentiments from Twitter and Delta wave data show that people recognize the benefits of taking the vaccine, which is better than being infected with Delta variant COVID-19.

At the outset, we asked: what is the state of the incorporation of sentiment analysis in epidemiological modeling? What are its advantages and limitations?

Epidemiological modeling that includes gleaning rationale for behavioral choices, such as vaccine hesitancy, from public sentiment analysis of social media channels appears to be in the Rogers Innovator stage but promises further adoption as efficacy improves [90]. Working in favor of more rapid diffusion, social media offers a rich set of real-time data that data mining may use to uncover changes in human behavior more quickly than traditional data-collection methods. An obvious challenge will be data validation, though even hoaxes promoted on the Internet can result in adverse social behavior in the short term [91]. Early identification of behavior—whether based on fact or fiction—will help models to rapidly adapted to reflect that change and provide information to responsible authorities to counter falsehoods and inappropriate behavior. Similarly, the early identification of behavioral changes may contribute to improved communications, protocols, and programs promoted by governments, organizations, societies, or individuals. In the longer term, improved model efficacy can speed the implementation of appropriate and timely interventions and reduce or delay the disease spread.

Sentiment analysis limitations include closed nations and closed networks [19] as access to data and demographics may limit the scope of the analysis or make it problematic. Segmentation of the population across an ever-increasing number of social media choices may drive the need to use multiple social media channels as well as the use of traditional data-collection methods to improve the prediction efficacy [92]. For vaccination modeling or promotion, simple GPS tracing or monitoring one or even two social media channels such as Facebook, Twitter, or Google will not reach the levels of model efficacy or vaccination compliance desired. Varying levels of demographic engagement in social media may bias results. For example, in the United States of America, more than 67% of people aged 65 or older are the demographic most at risk to the virus and are consequently the most fully vaccinated [93]. Identifying vaccine and booster hesitancy factors within this demographic through social media is limited by the fact that 25% of the older demographic does not even use the Internet [94]. Thus, successful data-mining and analysis of social media channels for this 25% will in all likelihood be limited to means other than social media. Since the level of threat from the virus varies by age, a very large number of younger Americans are deferring or even foregoing vaccination, possibly feeling that potential side effects of the vaccine are a greater threat than the threat of the virus [95]. Thus, future comprehensive research must be cognizant of the radical differences in behavior between segments of the population and plan a data collection methodology that spans complementary social media channels as well as incorporates non-traditional data-collection methods.

## 6. Conclusions

Integrating social media into disease prediction models and health protocols models give promising findings that support this trend of studies. During the review, we found studies that encourage using social media tools as rich data sources and behavioral modification tools that help in the decision-making process. To illustrate this, social media applications are considered as significant factors to provide valid data that improve the models’ inputs drastically. In addition, the media has a strong contribution to control public behavior and change it to be healthier through awareness programs. Moreover, this smart cooperation can save time, increase the accuracy of predictions, and reduce or delay the disease spread. We classified the epidemiological models into five categories: Epidemiological State Model, Epidemiological Statistical Forecast Models, Theoretical intervention approaches and models, Agent-based and Multiagent systems modeling, and Artificial Intelligence and Hybrid models. We explored features and limitations of each type and their contribution to the epidemiological modeling field. We then described whether the models were integrated with social media and/or social networks factors in previous studies.

Increasing public confidence in the models, protocols, and policies infers a more stable and compliant behavior pattern that, if scientifically based, will decrease the pandemic spread, promote public health protocols, and support vaccine implementation plans. For future pandemic modeling and analysis, inconsistent national and state health practices, and compounding international and intranational demographical divergent beliefs and behaviors, we conclude that various forms of agent modeling combined with social and traditional media data sourcing will produce the most efficacious models and, hence, the most efficacious health protocols.

## Data Availability

Not applicable.

## Abbreviation

SIR	Susceptible, Infected, Recovered
SIS	Susceptible, Infected, Susceptible
SIRD	Susceptible, Infected, Recovered, Deceased
MSIR	Maternally derived immunity, Susceptible, Infected, Recovered
SEI	Susceptible, Exposed, Infected
SEIR	Susceptible, Exposed, Infected, Recovered
SEIS	Susceptible, Exposed, Infected, Susceptible
MSEIR	Maternally derived immunity, Susceptible, Exposed, Infected, Recovered
MSEIRS	Maternally derived immunity, Susceptible, Exposed, Infected, Recovered, Susceptible
SLIRDS	Susceptible-Latent-Infected-Recovered-Dead-Susceptible
EIH	Exposed, Infected, Hospitalized
SIHR	Susceptible, Infected, Hospitalized, Recovered

## Appendix A

The below table summarizes the presented models in the manuscript. * Social Media: SM; * Social Network: SN.

**References**	**How Was SM or SN Used?**	**Platform/** **Application?**	**Calibrated with SM or SN?**	**Goal/Predict no of?**	**Type**	**Model**
[96]	-----	-----	No	Susceptible cases Infected cases Recovered cases	Epidemiological State models	Susceptible, Infected, Recovered (SIR)
[97]	-----	-----	No	Susceptible cases infected cases	Epidemiological State models	Susceptible, Infected, Susceptible (SIS)
[98]	-----	-----	No	Susceptible cases infected cases Recovered cases Deaths cases	Epidemiological State models	Susceptible, Infected, Recovered, Deceased (SIRD)
[99]	-----	-----	No	Susceptible cases infected cases Recovered cases	Epidemiological State models	Maternally derived immunity, Susceptible, Infected, Recovered (MSIR)
[82]	Use media to modify public behavior	Media awareness programs	Yes/SM	Exposed cases Infected cases Recovered cases	Epidemiological State models	Susceptible, Exposed, Infected (SEI)
[100]	-----	-----	No	Susceptible cases Exposed cases infected cases Recovered cases	Epidemiological State models	Susceptible, Exposed, Infected, Recovered (SEIR)
[101]	-----	-----	No	Susceptible cases Exposed cases infected cases	Epidemiological State models	Susceptible, Exposed, Infected, Susceptible (SEIS)
[102]	-----	-----	No	Susceptible cases Exposed cases infected cases Recovered cases	Epidemiological State models	Maternally derived immunity, Susceptible, Exposed, Infected, Recovered (MSEIR)
[103]	-----	-----	No	Susceptible cases Exposed cases infected cases Recovered cases	Epidemiological State models	Maternally derived immunity, Susceptible, Exposed, Infected, Recovered, Susceptible (MSEIRS)
[104]	-----	-----	No	Susceptible cases Latent cases Infected cases Recovered cases Deaths cases	Epidemiological State models	Susceptible-Latent-Infected-Recovered-Dead-Susceptible (SLIRDS)
[105]	Use media to modify public behavior	Media awareness programs	Yes/SM	Exposed cases Infected cases Hospitalized cases	Epidemiological State models	Exposed, Infected, Hospitalized (EIH)
[78]	Use media to modify public behavior	Media awareness programs	Yes/SM	Susceptible cases Infected cases Hospitalized cases Recovered cases	Epidemiological State models	Susceptible, Infected, Hospitalized, Recovered (SIHR)
[106]	-----	-----	No	Infected cases Hospitalized cases Deaths cases	Epidemiological Statistical Forecast Models	Differential Equations Leads to Predictions of Hospitalizations and Infections (DELPHI);
[83]	Data source	Google and Twitter	Yes/SM	Infected cases	Epidemiological Statistical Forecast Models	Auto regressive integrated moving average (ARIMA)
[61]	-----	-----	No	Infected cases Death cases Predict time of pandemic peak	Epidemiological Statistical Forecast Models	Los Alamos National Laboratory COVID-19 forecasting using Fast Evaluation and Estimation (LANL COFFEF)
[107]	-----	-----	No	“Forecast how likely a patient’s disease is to worsen while being treated in a hospital and at what point in their care that might happen”	Epidemiological Statistical Forecast Models	John Hopkins model COVID-19 prediction (JHU COVID-19 prediction)
[20]	-----	-----	No	Susceptible cases Exposed cases Infected cases Quarantined cases Recovered cases Vaccinated cases	Epidemiological Statistical Forecast Models	Susceptible, Exposed, Infected, Quarantined, Recovered, Dead, Vaccinated forecasting (SEIQRDV.F).
[26]	Data source	Facebook	Yes/SM	Promote population to follow healthy behavior Predict changes in health behaviors of individuals	Theoretical Interventions model	Health Belief Model (HBM)
[27]	-----	-----	No	Predict the human behavior	Theoretical Interventions model	The Theory of Planned Behavior (TPB)
[28]	-----	-----	No	Explains how individuals are motivated to act to protect themselves	Theoretical Interventions model	Protection Motivation Theory (PMT)
[51]	-----	-----	No	Modeling disease dynamics and fear as two interacting contagion processes	Agent-based model	The Coupled Contagion Dynamics of Fear and Disease (CCDFD) model
[52]	-----	-----	No	Testing effects of different levels of social distancing policies on the diseases spread	Agent-based model	The Social Distancing (SD) model
[53]	-----	-----	No	Project epidemic trends Explore intervention scenarios Estimate resource needs.	Agent-based model	COVID-19 Agent-based Simulator (COVASIM) model
[54]	-----	-----	No	Simulate the epidemiological and economic impacts of social distancing policies	Agent-based model	COVID-19 agent-based simulation (COVID-ABS) model
[55]	-----	-----	No	“Effectiveness of a nationwide vaccine campaign in response to different vaccine efficacies, the willingness of the population to be vaccinated, and the daily vaccine capacity under two different federal plans”. Studying the interactions between nonpharmaceutical interventions and vaccines	Agent-based model	COVID-19 Agent-based Simulator (COVASIM) and Vaccination model
[50]	-----	-----	No	Susceptible cases Infected cases Recovered cases Quarantine impact Transport restrictions impact Effectiveness of the interventions on the disease spread	Multiagent system model	DMAS-SIR model
[57]	Data source	Mobile phones-Calls	Yes/SN	Trace users’ phones and their mobility through network to study effects of government’ interventions on virus spread	Agent-based model	Frias-Martinez model (FM)
[56]	Data source	Mobile phones-GPS traces	Yes/SN	Trace users’ phones and their mobility through GPS to study effects of government’ interventions on virus spread	Agent-based model	University of Texas at Austin’s (UT COVID-19-Social distancing) model
[59]	-----	-----	No	Infected cases Deaths cases	Artificial Intelligence and Hybrid models	Y Youyang Gu COVID-19 (YYG) model
[61]	-----	-----	No	Processing population’ images to detect who wear mask or who not	Artificial Intelligence and Hybrid models	Deep transfer learning (DTL) model
[62,63]	Data source	Mobile phones-GPS	Yes/SN	Effectiveness of the interventions on the disease spread No of required beds and at hospitals and care units Trace users’ phones and their mobility through GPS	Artificial Intelligence and Hybrid models	University of Virginia Biocomplexity Center PatchSim COVID-19 (UVA COVID-19)
[66]	Data source	Mobile phones-GPS	Yes/SN	Effectiveness of the interventions on the disease spread Trace users’ phones and their mobility through GPS	Artificial Intelligence and Hybrid models	Institute for Health Metrics and Evaluation COVID-19 (IHME COVID-19)
[67]	-----	-----	No	Infected cases Deaths cases No of required beds and at hospitals and care units	Artificial Intelligence and Hybrid models	Massachusetts Institute of Technology COVID-19 (MIT University COVID-19) model
[87]	Data source	Twitter/Users’ tweets	Yes/SM	Study and analyze Twitter users’ opinions, beliefs, and emotions about vaccination	Artificial Intelligence and Hybrid models	Twitter vaccination analysis (TWVA) model
